# Ten-year trends in overweight and obesity in the adult Portuguese population, 1995 to 2005

**DOI:** 10.1186/1471-2458-11-772

**Published:** 2011-10-07

**Authors:** Pedro Marques-Vidal, Fred Paccaud, Paula Ravasco

**Affiliations:** 1 Institute of Social and Preventive Medicine (IUMSP), CHUV and Faculty of biology and medicine, Lausanne, Switzerland; 2 Unidade de Nutrição e Metabolismo, Instituto de Medicina Molecular da Faculdade de Medicina da Universidade de Lisboa, Portugal

## Abstract

**Background:**

There is little information regarding the trends in body mass index (BMI) and obesity in the overall Portuguese population, namely if these trends are similar according to educational level. In this study, we assessed the trends in the prevalence of overweight and obesity in the Portuguese population, overall and by educational level.

**Methods:**

Cross-sectional national health interview surveys conducted in 1995-6 (n = 38,504), 1998-9 (n = 38,688) and 2005-6 (n = 25,348). Data were derived from the population and housing census of 1991 and two geographically-based strata were defined. The sampling unit was the house, and all subjects living in the sampling unit were surveyed. Height and weight were self-reported; the effects of gender, age group and educational level were also assessed by self-reported structured questionnaires. Bivariate comparisons were performed using Chi-square or analysis of variance (ANOVA). Trends in BMI levels were assessed by linear regression analysis, while trends in the prevalence of obesity were assessed by logistic regression.

**Results:**

Mean (±standard deviation) BMI increased from 25.2 ± 4.0 in 1995-6 to 25.7 ± 4.5 kg/m^2 ^in 2005-6. Prevalence of overweight remained stable (36.1% in 1995-6 and 36.4% in 2005) while prevalence of obesity increased (11.5% in 1995-6 and 15.1% in 2005-6). Similar findings were observed according to age group. Mean age-adjusted BMI increase (expressed in kg/m^2^/year and 95% confidence interval) was 0.073 (0.062, 0.084), 0.016 (0.000, 0.031) and 0.073 (0.049, 0.098) in men with primary, secondary and university levels, respectively; the corresponding values in women were 0.085 (0.073, 0.097), 0.052 (0.035, 0.069) and 0.062 (0.038, 0.084). Relative to 1995-6, obesity rates increased by 48%, 41% and 59% in men and by 40%, 75% and 177% in women with primary, secondary and university levels, respectively. The corresponding values for overweight were 6%, 1% and 23% in men and 5%, 7% and 65% in women.

**Conclusion:**

Between 1995 and 2005, obesity increased while overweight remained stable in the adult Portuguese population. Although higher rates were found among lesser educated subjects, the strong increase in BMI and obesity levels in highly educated subjects is of concern.

## Backgound

Obesity is now a pandemic condition, affecting approximately half a billion people worldwide [1]. Portugal is a South European country characterized by a high prevalence of overweight (39.4% to 41.8%) and obesity (14.2% to 21.3%) [2,3], ranking among the highest in Europe [4] which has prompted the government to launch a nationwide obesity prevention program [5,6]. There is no population-based data regarding overweight and obesity before 1995, but two studies conducted on male conscripts (mean age 18 years) showed an increase in overweight and obesity from 8.1% in 1960 to 18.0% in 1990 in the Lisbon region [7] and from 10.5% in 1986 to 21.3% in 2000 in Portugal [8]. In previous studies, we have shown that the prevalence of overweight and obesity were increasing in the Portuguese population [9] and that, in Switzerland, this increase in the prevalence of obesity was larger in low education strata of the population [10], a finding also observed in a prospective study conducted in an urban Portuguese population [11]. Still, to our knowledge, no information regarding these trends according to educational levels has ever been published for the overall Portuguese population. As a lower educational level is associated with worse health behaviours [12-14] and to a lower awareness of the health impact of excess weight [15,16] it is important to assess the impact of education on obesity levels and trends.

In this study, we assessed the 10-year trends in the prevalence of overweight and obesity in the Portuguese population, overall and by educational level, using data from the Portuguese National Health Surveys. The information collected will allow a better allocation of resources aimed at preventing the obesity epidemic in Portugal, by targeting specific groups and/or tailoring the preventive messages.

## Methods

### National Interview Surveys

The Portuguese National Health Survey is an official survey conducted by the Portuguese National Institute of Statistics in collaboration with the National Health Observatory under the responsibility of the Portuguese Ministry of Health. The National Health Survey provides information for the planning of the Portuguese health system and also to the WHO, Eurostat and OECD. The survey is anonymous and as part of the official statistics no approval from an ethics committee is necessary.

The methodology of the Portuguese national interview surveys has been described previously [9,17]. Briefly, the National Health Surveys were conducted between May 1995 and April 1996 (1995-6), October 1998 and September 1999 (1998-9) and February 2005 and January 2006 (2005-6). The sampling frame was built on census data and included all subjects living in individual housing during that period (collective housing such as hospitals, prisons, military barracks, or retirement houses were excluded). The sample was considered representative of the main regions of continental Portugal (North, Center, Lisbon region, Alentejo, and Algarve). In the 2005-6 survey the autonomous regions of the Azores and Madeira were also included, but not considered in the present analysis. The primary sampling unit (PSU) was the house, and data were derived from the population and housing census. Within each main region, two strata were defined: the freguesias (corresponding to counties) and, within the freguesias, geographically defined units of ± 300 lodgings (240 in 2005-6). The PSUs were then randomly selected within each geographically defined unit. All subjects living in the sampling unit (house) were surveyed. All surveys were carried out in compliance with the Helsinki Declaration. Data were collected by trained staff according to a standardized protocol [17], and quality control was maintained by reapplying (by a different interviewer) the same questionnaire to 10% of the initial sample. Participation rates (defined as the percentage of households who responded) as reported by the National Institute of Statistics were 88% in 1995-6, 82% in 1998-9 and 76% in 2005-6. In the 2005-6 survey, participation rates were 83% for North, 76% for center, 70% for Lisbon and Tagus valley and 78% for Alentejo and Algarve; no data was available for the previous surveys. Sample size was proportional to the population of each region in the 95-96 and 98-99 surveys, which was not exactly the case in the 2005-6 survey. As sampling weights were only available for the 2005-6 survey [17], the original (unweighted) data was analyzed.

Data were provided upon request by the National Health Observatory (http://www.onsa.pt) (1995-6 and 1998-9) and the National Institute of Statistics (http://www.ine.pt) (2005-6). In 1995-6, 49,718 subjects were surveyed, of whom 38,504 (18,053 men and 20,451 women) were aged over 18 years and had reported height and weight measurements; the corresponding figures for 1998-9 were 48,606 and 38,688 (18,132 men and 20,556 women), respectively, and for 2005-6 they were 29,908 and 25,348 (12,026 men and 13,322 women).

### Data Collection

All data were obtained by interview. Height and weight were self-reported; the correlations between self-reported and measured height, weight and BMI values are usually high, with correlation coefficients >0.9 [18,19]. Overweight was defined as a BMI ≥25 and <30 kg/m2; obesity was defined as a BMI ≥30 kg/m^2 ^[20]. As self-reporting leads to an underestimation of obesity levels, several correction methods were used as in a previous study [10]: 1) a second threshold of ≥29.2 kg/m2 to define obesity [21], 2) age and-gender specific corrections for height and weight as reported in a Portuguese study [22] 3) gender-specific corrections for height and weight [23] and 4) gender-specific BMI corrections of 0.8 kg/m2 and 1.1 kg/m2 in men and women, respectively [23]. The proposed corrections are within the values reported in a review of the literature [24]. Although self-reported BMI underestimates the true prevalence of obesity in a population, still it has been considered as valid to be used in epidemiological studies [25] to assess trends [23] and to compare between countries [4].

Educational level was assessed by the number of years spent in school and classified into three groups: 6 years or less (primary school); 7 to 12 years (secondary school); and >12 years (university level). For the 1995-6 and 1998-9 surveys, age was provided as a continuous variable. For security reasons, it was not possible to obtain individual ages for the 2005-6 survey; hence, 10-year age groups were used. As the questionnaire on physical activity changed completely between 98-99 and 2005-6 [17], it was not possible to adjust for physical activity.

### Statistical analyses

Statistical analyses were conducted using SAS Enterprise Guide v.4.2 (SAS Inc, Cary, NC, USA). Results were expressed as mean ± standard deviation, as number of subjects and (percentage) or as percentage and (95% confidence interval) for prevalence. Bivariate comparisons were performed using Chi-square or analysis of variance (ANOVA). Yearly trends in BMI levels were assessed by bivariate or multivariate linear regression analysis and the results were expressed as slope (95% confidence interval). Multivariate analyses were performed using logistic regression for qualitative variables and a general linear model for quantitative variables; the corresponding results were expressed as Odds-ratio (OR) and [95% confidence interval] or as adjusted mean ± standard error. Statistical significance was considered for p < 0.05.

## Results

### Samples' characteristics

The main characteristics of the samples according to survey year are summarized in Table 1. Educational level as well as the percentage of subjects aged over 75 increased, while no differences were found regarding gender distribution.

**Table 1:** Characteristics of the participants and prevalence of overweight and obesity, overall and by gender

	1995-6	1998-9	2005-6	Test (p-value)
Sample size	38,504	38,688	25,348	
Women (%)	20,451 (53.1)	20,556 (53.1)	13,322 (52.6)	2.47 (0.30)
Age group (%)				
18-24	4,962 (12.9)	4,685 (12.1)	3,222 (12.7)	146.3 (<0.001)
25-34	5,389 (14.0)	5,722 (14.8)	3,309 (13.1)	
35-44	6,319 (16.4)	6,344 (16.4)	3,934 (15.5)	
45-54	6,319 (16.4)	6,379 (16.5)	4,249 (16.8)	
55-64	6,445 (16.7)	6,122 (15.8)	3,906 (15.4)	
65-74	5,567 (14.5)	5,707 (14.8)	3,917 (15.5)	
75+	3,503 (9.1)	3,729 (9.6)	2,811 (11.1)	
Educational level (%)				
Primary	28,570 (74.2)	26,682 (69.0)	15,060 (59.4)	1557.3 (<0.001)
Secondary	7,293 (18.9)	8,787 (22.7)	7,472 (29.5)	
University	2,641 (6.9)	3,219 (8.3)	2,816 (11.1)	
BMI (kg/m^2^)				
Average ± SD	25.2 ± 4.0	25.4 ± 4.1	25.7 ± 4.5	87.9 (<0.001)
Median (IQR)	24.8 (22.5 - 27.5)	25.0 (22.6 - 27.7)	25.2 (22.6 - 28.1)	
BMI categories (%)				
Normal	20,164 (52.4)	19,420 (50.2)	12,294 (48.5)	202.4 (<0.001)
Overweight	13,909 (36.1)	14,312 (37.0)	9,233 (36.4)	
Obese	4,431 (11.5)	4,956 (12.8)	3,821 (15.1)	
**Men**				
BMI (kg/m^2^)				
Average ± SD	25.4 ± 3.5	25.7 ± 3.7	25.9 ± 4.1	61.8 (<0.001)
Median (IQR)	25.1 (23.1 - 27.5)	25.4 (23.2 - 27.7)	25.6 (23.3 - 28.3)	
BMI categories (%)				
Normal	8,887 (49.2)	8,358 (46.1)	5,330 (44.3)	138.4 (<0.001)
Overweight	7,310 (40.5)	7,702 (42.5)	5,006 (41.6)	
Obese	1,856 (10.3)	2,072 (11.4)	1,690 (14.1)	
**Women**				
BMI (kg/m^2^)				
Average ± SD	25.0 ± 4.4	25.2 ± 4.5	25.4 ± 4.8	32.8 (<0.001)
Median (IQR)	24.4 (21.9 - 27.5)	24.7 (22.0 - 27.9)	24.8 (22.0 - 28.1)	
BMI categories (%)				
Normal	11,277 (55.1)	11,062 (53.8)	6,964 (52.3)	80.18 (<0.001)
Overweight	6,599 (32.3)	6,610 (32.2)	4,227 (31.7)	
Obese	2,575 (12.6)	2,884 (14.0)	2,131 (16.0)	

Obesity was defined as a body mass index ≥30 kg/m2. Results are expressed as number of participants and (percentage) or as mean ± standard deviation. IQR: interquartile range. Statistical analysis by chi-square or analysis of variance.

### Trends in overweight and obesity

Mean and median BMI as well as prevalence of obesity increased in both genders, while the prevalence of overweight remained relatively constant (Table 1). After adjusting for age group and educational level, BMI increased from 24.5 ± 0.1 kg/m^2 ^(adjusted mean ± standard error) in 1995-6 to 25.1 ± 0.1 kg/m^2 ^in 2005-6 (25.1 ± 0.1 to 25.7 ± 0.1 kg/m^2 ^in men and 23.9 ± 0.1 to 24.6 ± 0.1 kg/m^2 ^in women).

Prevalence of overweight and obesity increased with age in both genders, but while obesity levels increased with time in almost all age groups, overweight levels remained relatively stable (Table 2). The prevalence of overweight and obesity was also higher among subjects with primary education, while subjects with secondary or university levels had rather comparable rates (Table 3). In men, the age-adjusted increases in mean BMI (expressed in kg/m^2^/year and 95% confidence interval) were 0.073 (0.062, 0.084), 0.016 (0.000, 0.031) and 0.073 (0.049, 0.098) in subjects with primary, secondary and university levels, respectively; the corresponding values in women were 0.085 (0.073, 0.097), 0.052 (0.035, 0.069) and 0.062 (0.038, 0.084). Obesity rates increased with time in all educational groups, while overweight levels increased mostly if not exclusively in the better educated subjects (Table 3). Relative to 1995-6, in 2005-6 obesity rates increased by 48%, 41% and 59% in men and by 40%, 75% and 177% in women with primary, secondary and university levels, respectively. The corresponding values for overweight were 6%, 1% and 23% in men and 5%, 7% and 65% in women.

**Table 2:** Prevalence of overweight and obesity, by survey year, sex and age group

Gender/age group	1995-6	1998-9	2005-6
**Men**			
Overweight (%)			
18-24	20.9 (19.4 - 22.5)	20.5 (18.9 - 22.1)	16.8 (15.0 - 18.6)
25-34	36.1 (34.3 - 37.9)	36.6 (34.8 - 38.4)	39.7 (37.4 - 42.1)
35-44	44.4 (42.6 - 46.1)	46.9 (45.1 - 48.7)	46.0 (43.7 - 48.2)
45-54	49.9 (48.1 - 51.7)	49.7 (47.9 - 51.4)	47.4 (45.2 - 49.6)
55-64	46.9 (45.0 - 48.7)	49.9 (48.0 - 51.7)	48.3 (46.0 - 50.6)
65-74	43.6 (41.7 - 45.5)	47.9 (46.0 - 49.8)	47.2 (44.9 - 49.6)
75+	37.8 (35.3 - 40.4)	42.7 (40.2 - 45.2)	43.8 (41.0 - 46.6)
Obesity (%)			
18-24	2.1 (1.5 - 2.6)	3.2 (2.5 - 3.9)	3.7 (2.8 - 4.6)
25-34	6.6 (5.6 - 7.5)	7.1 (6.1 - 8.0)	8.3 (7.0 - 9.6)
35-44	11.1 (10.0 - 12.3)	11.8 (10.7 - 13.0)	13.9 (12.3 - 15.4)
45-54	13.4 (12.2 - 14.6)	15.0 (13.7 - 16.3)	19.5 (17.7 - 21.2)
55-64	15.7 (14.4 - 17.0)	16.2 (14.8 - 17.6)	20.1 (18.3 - 22.0)
65-74	13.0 (11.7 - 14.3)	14.9 (13.5 - 16.3)	17.7 (15.9 - 19.5)
75+	7.8 (6.4 - 9.2)	10.1 (8.6 - 11.7)	12.9 (11.0 - 14.7)
**Women**			
Overweight (%)			
18-24	10.7 (9.4 - 11.9)	10.4 (9.1 - 11.7)	10.2 (8.6 - 11.7)
25-34	23.1 (21.5 - 24.7)	21.9 (20.4 - 23.4)	21.5 (19.5 - 23.4)
35-44	34.1 (32.4 - 35.7)	32.1 (30.5 - 33.7)	28.5 (26.6 - 30.5)
45-54	39.0 (37.4 - 40.7)	38.9 (37.2 - 40.5)	37.0 (35.0 - 39.0)
55-64	41.1 (39.4 - 42.7)	41.1 (39.4 - 42.7)	42.4 (40.3 - 44.6)
65-74	39.9 (38.1 - 41.6)	39.7 (38.0 - 41.4)	41.8 (39.7 - 43.8)
75+	29.3 (27.4 - 31.2)	33.5 (31.6 - 35.5)	32.8 (30.4 - 35.1)
Obesity (%)			
18-24	2.0 (1.4 - 2.5)	2.3 (1.6 - 2.9)	2.7 (1.9 - 3.5)
25-34	5.6 (4.7 - 6.4)	6.4 (5.5 - 7.3)	8.0 (6.6 - 9.3)
35-44	11.4 (10.3 - 12.4)	11.9 (10.8 - 13.0)	13.5 (12.0 - 14.9)
45-54	17.8 (16.5 - 19.1)	19.4 (18.1 - 20.7)	20.5 (18.8 - 22.1)
55-64	19.0 (17.7 - 20.3)	20.3 (18.9 - 21.7)	22.8 (21.0 - 24.6)
65-74	16.1 (14.8 - 17.4)	19.3 (17.9 - 20.7)	22.6 (20.8 - 24.4)
75+	11.7 (10.3 - 13.0)	14.2 (12.7 - 15.6)	16.6 (14.8 - 18.4)

Overweight was defined as a body mass index (BMI) ≥25 and <30 kg/m^2^; obesity was defined as a BMI ≥30 kg/m^2^. Results are expressed as percentage and (95% confidence interval).

**Table 3:** Prevalence of overweight and obesity, by survey year, sex and educational group

Gender/age group	1995-6	1998-9	2005-6
**Men**			
Overweight (%)			
Primary	42.9 (42.0 - 43.7)	45.7 (44.8 - 46.6)	45.5 (44.4 - 46.7)
Secondary	35.4 (33.9 - 36.9)	36.4 (35.0 - 37.8)	35.6 (34.1 - 37.1)
University	31.1 (28.5 - 33.7)	34.4 (31.9 - 36.9)	38.3 (35.6 - 41.1)
Obesity (%)			
Primary	11.8 (11.2 - 12.3)	13.6 (13.0 - 14.2)	17.5 (16.6 - 18.3)
Secondary	6.6 (5.8 - 7.3)	7.6 (6.8 - 8.3)	9.3 (8.4 - 10.2)
University	6.1 (4.8 - 7.5)	5.0 (3.8 - 6.1)	9.7 (8.0 - 11.3)
**Women**			
Overweight (%)			
Primary	36.9 (36.2 - 37.7)	37.8 (37.0 - 38.5)	38.7 (37.7 - 39.8)
Secondary	20.1 (18.7 - 21.4)	20.3 (19.1 - 21.5)	21.6 (20.3 - 22.9)
University	11.6 (9.9 - 13.2)	14.8 (13.2 - 16.4)	19.1 (17.2 - 21.1)
Obesity (%)			
Primary	15.3 (14.7 - 15.9)	17.8 (17.2 - 18.4)	21.4 (20.5 - 22.3)
Secondary	4.8 (4.1 - 5.5)	5.6 (4.9 - 6.3)	8.4 (7.4 - 9.3)
University	2.2 (1.4 - 2.9)	3.1 (2.3 - 3.8)	6.1 (4.9 - 7.2)

Overweight was defined as a body mass index (BMI) ≥25 and <30 kg/m^2^; obesity was defined as a BMI ≥30 kg/m^2^. Results are expressed as percentage and (95% confidence interval).

Multivariate logistic regression showed that the likelihood of presenting with obesity was significantly higher in 2005-6 compared to 1998-9 and 1995-6 in both genders. After adjusting for survey year, and age group, men with primary or secondary education had a significantly higher likelihood of presenting with obesity (1.79 and 1.30 fold higher likelihood, respectively) than subjects with university level. Women with primary or secondary education also had a higher likelihood of presenting with obesity (3.80 and 1.76 fold higher likelihood, respectively) than women with university level (Table 4). Similar findings were obtained when the analysis was applied to overweight, i.e. a higher likelihood of presenting with overweight among lesser educated subjects (Table 5).

**Table 4:** Multivariate analysis of the determinants of obesity in the Portuguese population, by gender

	Men	Women
**Survey year**		
1995-6	1 (ref.)	1 (ref.)
1998-9	1.15 [1.07 - 1.23]	1.18 [1.11 - 1.25]
2005-6	1.51 [1.41 - 1.62]	1.49 [1.40 - 1.59]
**Educational level (%)**		
Primary	1.79 [1.57 - 2.05]	3.80 [3.25 - 4.43]
Secondary	1.30 [1.13 - 1.50]	1.76 [1.49 - 2.09]
University	1 (ref.)	1 (ref.)
**Age group (%)**		
18-24	1 (ref.)	1 (ref.)
25-34	2.43 [2.05 - 2.88]	2.40 [1.98 - 2.91]
35-44	4.11 [3.50 - 4.82]	4.19 [3.49 - 5.03]
45-54	5.30 [4.53 - 6.21]	6.41 [5.35 - 7.68]
55-64	5.67 [4.84 - 6.66]	6.37 [5.31 - 7.63]
65-74	4.75 [4.04 - 5.59]	5.56 [4.63 - 6.68]
75+	2.97 [2.48 - 3.56]	3.78 [3.13 - 4.57]

Statistical analysis by logistic regression. Results are expressed as Odds-ratio and [95% confidence interval]. The results for each variable are adjusted for the other two. Obesity was defined as a body mass index ≥30 kg/m^2^.

**Table 5:** Multivariate analysis of the determinants of overweight in the Portuguese population, by gender

	Men	Women
Survey year		
1995-6	1 (ref.)	1 (ref.)
1998-9	1.14 [1.09 - 1.19]	1.07 [1.03 - 1.12]
2005-6	1.20 [1.14 - 1.26]	1.20 [1.14 - 1.26]
Educational level (%)		
Primary	1.45 [1.34 - 1.56]	3.12 [2.86 - 3.40]
Secondary	1.26 [1.16 - 1.36]	1.59 [1.45 - 1.74]
University	1 (ref.)	1 (ref.)
Age group (%)		
18-24	1 (ref.)	1 (ref.)
25-34	2.55 [2.36 - 2.76]	2.21 [1.99 - 2.44]
35-44	4.05 [3.74 - 4.37]	3.64 [3.31 - 4.01]
45-54	5.11 [4.72 - 5.54]	5.20 [4.72 - 5.74]
55-64	4.99 [4.59 - 5.42]	5.59 [5.06 - 6.17]
65-74	4.19 [3.85 - 4.56]	4.87 [4.40 - 5.38]
75+	2.98 [2.71 - 3.27]	2.83 [2.55 - 3.15]

Statistical analysis by logistic regression after excluding subjects with obesity. Results are expressed as Odds-ratio and [95% confidence interval]. The results for each variable are adjusted for the other two. Obesity was defined as a body mass index ≥25 and <30 kg/m^2^.

## Discussion

Overall, our results indicate that between 1995 and 2005, obesity increased while overweight remained stable in the adult Portuguese population. These findings are partly in agreement with previous studies [1,2]. This selective increase in obesity levels is of concern, as the impact of obesity on disease is stronger than for overweight. Our results thus suggest that strong preventive measures are necessary to curb this worrying trend, and it will be of interest to know the final results of the National Plan to Fight Obesity (Programa Nacional de Luta contra a Obesidade), which ended in 2010 [5,6].

In the 2005-6 survey, more than half of the Portuguese population aged over 18 was overweight or obese, the rates being higher in men than in women. These values are in agreement with previously published studies for the Portuguese population which used objectively measured data. For instance, the uncorrected prevalence of obesity among subjects aged 18-64 in the 2005-6 survey was 14.0%, close to the 14.2% reported by a national study conducted in 2003-5 within the same age group [2]. Correcting for underestimation due to self-report led to even higher overweight and obesity levels, with almost two thirds (63%) of the Portuguese population being overweight or obese, and over one fifth (21.3%) being obese (Table 5), a figure identical to the prevalence of obesity reported in another study which also used measured data [3]. Hence, the overall (un)corrected prevalence of obesity reported in this study appear to be in agreement with the results from studies using objectively measured data. Although self-reported BMI underestimates the true prevalence of obesity in a population, still it can be used to assess trends [23] and to compare between countries [4], provided the same methodology of data collection is used between studies, which was here the case. Finally, although comparison with international data might be difficult, our data indicate that, in 2005-6, the overall prevalence of reported obesity in Portugal was higher than in Switzerland but comparable to France (Table 6).

**Table 6:** Prevalence of uncorrected, reported obesity in Switzerland and other countries, by age group

Period	Portugal 2005-6	Switzerland, 2007	France, 2006	France, 2009
Men				
[18-34]	6.0	4.9	7.5 ^a^	8.5 ^a^
[35-44]	13.9	7.8	11.5	13.0
[45-54]	19.5	10.3	15.0	16.0
[55-64]	20.1	13.4	19.0	20.0
[65-74]	17.7	13.3	17.0	18.0
[75+	12.9	9.2		
Women				
[18-34]	5.5	4.6	10.0 ^a^	12.0 ^a^
[35-44]	13.5	6.0	15.0	14.5
[45-54]	20.5	8.7	15.0	16.0
[55-64]	22.8	10.8	18.0	19.5
[65-74]	22.6	13.1	16.0	18.0
[75+]	16.6	9.9		

Obesity defined as a BMI ≥30 kg/m^2^. Data for Switzerland obtained from [10]; data from France obtained from [39]. ^a^: for age group 25-34 years. Results are expressed in percentage.

Contrary to obesity, overweight rates tended to stabilize or even to decrease slightly in the overall population. These findings do not replicate the ones from a previous study, which showed an increase in the prevalence of overweight in the Portuguese population from 35.2% in 1995-8 to 39.4% in 2003-5 [2], but the precise rationale for this stabilization is currently unknown. A possible explanation would be that the magnitude of the self-reporting bias for height and weight increased between 1995-6 and 2005-6 as noted in other studies [26], but we lack data to verify this hypothesis. This increase in self-reporting bias would have lessened the observed increase in BMI and obesity levels, and it is thus likely that these increases, if objectively measured, could have been even greater. Interestingly, mean and median BMI increased similarly during the study period, suggesting an overall shift of the BMI distribution rather than the possibility of an ever expanding tail.

Some studies have shown that not only lower educated subjects present with higher obesity levels, but also that this "obesity gap" is currently widening [10,27,28]. Still, other studies found no such widening gap [29,30]. In this study, the mean yearly increase in BMI levels was similar for subjects with primary and university level, while subjects with secondary education tended to present a lower yearly increase in BMI levels. Our findings are partly in agreement with a prospective study conducted in Northern Portugal [11] which showed a similar incidence of obesity irrespective of educational level in men, while a lower incidence was found in better educated women. The differences among women between our study and the one from Northern Portugal might partly be due to the fact that the former study was conducted in an urban setting while our data pertains to the whole country. The differences may also be related with the different methodology used to measure height and weight (self vs. measured) instated with the sample basis, since the reporting bias is higher in women than in men and is dependent of education level. Overall, our data confirm the presence of an "educational obesity gap" in the Portuguese population, but not it's widening. Several reasons for the similar increase in BMI levels among subjects with basic and university education can be proposed. For instance, Portuguese subjects with a university degree tend to exercise less [31] and to remain sit longer [32] than lower educated subjects; subjects with higher education also tend to have a lower quality diet [33] with a higher fat content [34]. Conversely, lower educated subjects are less likely to engage in weight control, to consider obesity as a disease and to consider weight loss as a health priority [15,16]. Hence, and contrary to what is observed in other countries [35,36], it seems that the highly educated Portuguese tend to adhere to unhealthy lifestyles leading to increased obesity rates. This strong increase in overweight prevalence rates among subjects with university calls for preventive measures, as it is possible that, in a not-so-far future, the increase in obesity levels will further accelerate in this group. The lower increase in BMI and prevalence rates among subjects with secondary education was unexpected and the reasons for such a difference await further investigation.

The major advantage of this study is that it is based in nationally representative samples, and that the data has been collected using the same methodology throughout time, thus enabling assessing trends with confidence. Still, this study also has several limitations worth mentioning. Namely, height and weight were self-reported, leading to an underestimation of obesity prevalence. Although we used a correction method, still the best issue would be to conduct a nationwide examination survey, which unfortunately is not programmed for the next years. Further, selective underreporting of weight and over reporting of height are influenced by actual body size as well as sociodemographic characteristics [37]. Therefore, it is possible that bias increases over time, given that the prevalence of obesity is also increasing, and this has an impact on the observed trend. The changes in educational level can also lead to selective bias, but its magnitude is difficult to assess, as it has been suggested that lesser educated people overestimate their height while better educated people underestimate their weight [37]. Hence, it is likely that the real trend is actually worse than observed, although the opposite has been suggested. Further, for the 2005-6 raw, unweighted data was used, which might not correspond exactly to the weight of each region. Still, the unweighted estimates for obesity (15.1%, 14.1% and 16.0% for overall, men and women, respectively) were quite similar to the weighted ones (15.2%, 14.3% and 16.0% for overall, men and women, respectively [17]). Hence, it does not appear that using unweighted data from the 2005-6 led to considerable differences in obesity prevalence estimates. Finally, as participation rates differed between regions, it is possible that the exact prevalence of overweight and obesity might be slightly different from the reported in this study. Still, as we could find no data regarding the regional participation rates of the previous studies, it is currently not possible to know if these participation rates changed during time and what might be their impact on the estimations provided. Nevertheless, it should be noted that these household participation rates were comparable to another study (75%) [38].

## Conclusion

Between 1995 and 2005, obesity increased while overweight remained stable in the adult Portuguese population. Taking into account underestimation due to self-reporting, more than half of the Portuguese population is currently overweight or obese. Although higher rates were found among lesser educated subjects, the strong increase in BMI and obesity levels in highly educated subjects is of concern.

## Declaration of competing interests

The authors report no competing interests.

## Authors' contributions

PMV designed the study, ran the statistical analysis and wrote part of the manuscript. PR obtained the data and wrote part of the manuscript. FP revised the manuscript critically for important intellectual content. All authors (PMV; PR and FP) have given final approval of the version to be published.

## Pre-publication history

The pre-publication history for this paper can be accessed here:

http://www.biomedcentral.com/1471-2458/11/772/prepub
